# How to improve scientific peer review: Four schools of thought

**DOI:** 10.1002/leap.1544

**Published:** 2023-04-27

**Authors:** Ludo Waltman, Wolfgang Kaltenbrunner, Stephen Pinfield, Helen Buckley Woods

**Affiliations:** ^1^ Centre for Science and Technology Studies (CWTS) Leiden University Leiden The Netherlands; ^2^ Research on Research Institute (RoRI) UK; ^3^ Information School University of Sheffield Sheffield UK

**Keywords:** innovation, peer review, scholarly publishing, school of thought

## Abstract

Peer review plays an essential role as one of the cornerstones of the scholarly publishing system. There are many initiatives that aim to improve the way in which peer review is organized, resulting in a highly complex landscape of innovation in peer review. Different initiatives are based on different views on the most urgent challenges faced by the peer review system, leading to a diversity of perspectives on how the system can be improved. To provide a more systematic understanding of the landscape of innovation in peer review, we suggest that the landscape is shaped by four schools of thought: The Quality & Reproducibility school, the Democracy & Transparency school, the Equity & Inclusion school, and the Efficiency & Incentives school. Each school has a different view on the key problems of the peer review system and the innovations necessary to address these problems. The schools partly complement each other, but we argue that there are also important tensions between them. We hope that the four schools of thought offer a useful framework to facilitate conversations about the future development of the peer review system.

**Key points**

- There are several different perspectives on how peer review can be improved.
- The landscape of innovation in peer review is shaped by four schools of thought: The Quality & Reproducibility school, the Democracy & Transparency school, the Equity & Inclusion school, and the Efficiency & Incentives school.
- Each school has a different view on the key problems in peer review and the innovations necessary to address these problems.
- While the schools partly complement each other, there are also important tensions between them.

## INTRODUCTION

Peer review is generally seen as one of the cornerstones of the scholarly publishing system. Although aware of its shortcomings, most researchers seem to be reasonably satisfied with the way peer review is organized and conducted (Mulligan et al., 2013; Nicholas et al., 2015). However, there are also outspoken critics. For instance, Richard Smith, former editor of *BMJ*, considers peer review to be ‘slow, expensive, profligate of academic time, highly subjective, something of a lottery, prone to bias, and easily abused’ (Smith, 2006). Recently, he even suggested that it may be ‘time for peer reviewers to rise up in rebellion’ (Smith, 2021). Peer review is also very costly. According to a conservative estimate, the ‘monetary value of the time US‐based reviewers spent on reviews was over 1.5 billion USD in 2020’ (Aczel et al., 2021). Evaluating and improving the organization of peer review is clearly of major importance.

A large number of studies have been performed to develop a better understanding of weaknesses of peer review, and many initiatives have been undertaken that aim to improve the system. We provide an overview of this work in two related papers. One paper presents a meta‐summary of the literature on innovations in peer review, collating the findings of a number of recent review articles (Woods et al., 2022). The other paper provides an overview of ongoing innovations in peer review based on a survey of publishers and other organizations active in scholarly publishing (Kaltenbrunner, Pinfield, et al., 2022).

As shown in our papers, the landscape of innovation in peer review is highly complex. A large variety of initiatives aimed at improving the peer review system have been developed, focusing on many different aspects of the system. These initiatives differ not only in how they set out to improve the system, but also in what they consider to be its key problems. Different initiatives are often based on different views of the most urgent challenges faced by the peer review system. In addition, there seem to be tensions between some of the initiatives, caused by incompatible ideas on the role of peer review in the broader system of scholarly publishing.

Innovations taking place in the scholarly communication system are occurring in a broad range of organizations and communities but are not necessarily evenly spread. There is evidence of some clustering of innovation in particular publishers and other scholarly communication organizations. As far as disciplines are concerned, certain communities have shown a willingness to innovate, for instance psychology in response to concerns about reproducibility, whilst others, such as many humanities disciplines, less so. Nevertheless, the developments we observe in this paper have wide applicability across organizations and communities, with many of them having the potential to be widely adopted.

In this paper, we aim to offer a more structured and systematic perspective on the landscape of innovation in peer review. Based on our related work (Kaltenbrunner, Pinfield, et al., 2022; Woods et al., 2022), we suggest that the landscape is shaped by four schools of thought: The Quality & Reproducibility school, the Democracy & Transparency school, the Equity & Inclusion school, and the Efficiency & Incentives school. Each school has a different view on the key problems of the peer review system and the innovations that are necessary to address these problems. By distinguishing between the four schools, we hope to provide a framework that facilitates conversations about the future development of the peer review system, in particular conversations between individuals and organizations that have different perspectives on the system. The framework that we present in this paper also addresses a recent call for more theory development in research on peer review (Hug, 2022).

Our work is restricted to peer review in the context of scholarly publishing. Peer review in other contexts, such as grant proposals and research assessment settings, falls outside the scope of our work. However, within the context of scholarly publishing, we adopt a broad perspective on peer review, covering not only the activities of researchers that act as reviewers, but also the work done by editors, publisher staff, and others that contribute to quality assurance of scientific work. The lines between all these different quality assurance activities and actors are often difficult to identify clearly, particularly as they vary across different models of peer review, and so it makes sense to think about peer review in this broad way. This is particularly the case since some of the developments we discuss in this paper are implicitly challenging what constitutes a ‘peer’ for the purposes of peer review, extending the definition in various ways (e.g., specialist statistical reviewers, impact specialists, expert patients, or researchers working in non‐academic settings), making it important to take these into account.

In their book chapter, *Open science: One term, five schools of thought*, Fecher and Friesike (2014) identify five schools of thought in the discourse on open science. Our suggestion to structure discussions about peer review innovation in terms of different schools of thought is loosely inspired by their work. However, there is no direct connection between the four peer review schools that we introduce in the present paper and the five open science schools distinguished by Fecher and Friesike. Like Fecher and Friesike, we do not consider the distinction between different schools of thought to be clear cut. Rather, we see schools of thought as broad categories within which there are key commonalities but across which there may also be tensions. We argue, nevertheless, that each school is representative of a specific perspective on peer review innovation and particular priorities in improving the peer review system.

**FIGURE 1:**
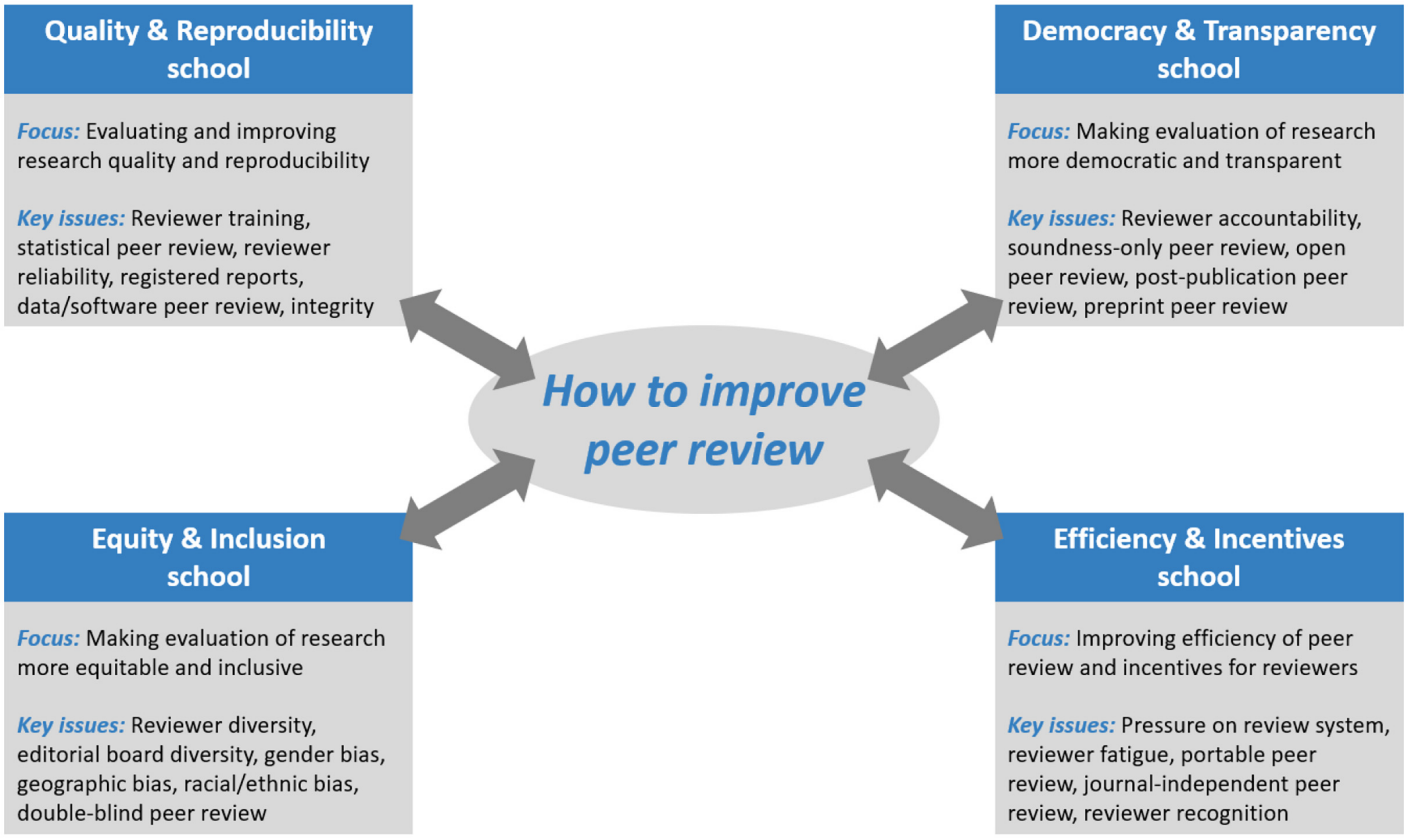
Four schools of thought, each offering a different perspective on the problems of the peer review system and the innovations needed to address these problems. For each school, a number of key issues of concern are presented. These issues merely serve as examples and do not reflect the full scope of a school.

Figure 1 summarizes some of the core characteristics of the Quality & Reproducibility school, the Democracy & Transparency school, the Equity & Inclusion school, and the Efficiency & Incentives school. We discuss the four schools of thought in more detail in Sections QUALITY & REPRODUCIBILITY SCHOOL, EFFICIENCY & INCENTIVES SCHOOL. For each school, we present its view on the key problems of the peer review system and ways in which these problems can be addressed. Building on our recent papers (Kaltenbrunner, Pinfield, et al., 2022; Woods et al., 2022), we offer examples of developments that we consider to be representative of the different schools, and we discuss concrete innovations resulting from these developments. Our aim is to provide a general understanding of the viewpoints of the different schools of thought. We do not aim to give an exhaustive overview of all the work done in the four schools. In Sections 6 and 7, we discuss how the different schools may complement each other, but also how there may be tensions between the schools. We present some concluding remarks in Section 8.

## QUALITY & REPRODUCIBILITY SCHOOL

The Quality & Reproducibility school focuses on the role peer review can play in ensuring published research is of a high quality and wherever possible reproducible. This school is interested in initiatives in peer review that improve the quality of the review process (e.g., improving review reports) in order to improve the quality of the published research itself. Examples of such innovations include reviewer training, use of checklists, addition of a statistical reviewer, revealing of reviewer identities, and anonymizing authors. Jefferson et al. (2007) and Bruce et al. (2016) provide reviews of studies that analyse quality improvements resulting from innovations in peer review in the biomedical sciences. Based on a systematic review and meta‐analysis of 22 randomized controlled trials, Bruce et al. draw conclusions in a variety of areas, including casting doubt on the value of training in improving quality of reviews or of review templates in improving the articles. They also find that ‘blinded peer review did not affect the quality of the peer review report or rejection rate’, but that statistical peer review did improve the quality of the paper. However, they conclude that the limitations of the evidence meant ‘we cannot provide conclusive recommendations on the use of interventions to improve quality of peer review … the state of the evidence falls short of generating empirical support’. Bruce et al. emphasize ‘the need for additional experimentation on this topic and exploration into the drivers of publication quality’.

The Quality & Reproducibility school is also concerned with the level of agreement between different peer reviewers of the same manuscript. A high level of agreement is often seen as evidence of the reliability of peer review, as discussed in a review of the relevant literature provided by Bornmann (2011). In a meta‐analysis of 48 studies, Bornmann et al. (2010) report ‘a low level of inter‐rater reliability’. This may indicate that peer reviewers are not able to reliably distinguish between lower‐quality and higher‐quality research. Many therefore consider the low level of agreement between peer reviewers to be problematic. It has led some to suggest that peer review resembles a ‘game of chance’ and that the number of reviewers per manuscript may need to be increased (Neff & Olden, 2006). However, as discussed by Bornmann (2011) and Lee et al. (2013), others do not necessarily regard a low level of agreement as undesirable, since it may result from different peer reviewers bringing in different types of expertise and focusing on different aspects of a manuscript. For instance, Bailar (1991) argues that ‘too much agreement is in fact a sign that the review process is not working well, that reviewers are not properly selected for diversity’.

Over the last decade, reproducibility and replicability of research has received a lot of attention, leading to a new perspective on the role of peer review. Registered reports, in which peer review of a research plan and in‐principle acceptance take place before carrying out data collection and analysis, have been introduced as a promising approach to improve the quality and reproducibility of research in the social sciences and the life sciences (Chambers & Tzavella, 2022). According to Soderberg et al. (2021), ‘the standard model of peer review and publication is vulnerable to biases that can reduce the credibility of the published literature’. These include preference being given to positive rather than negative results, novel rather than replication or incremental findings, and papers telling an unequivocal story rather than one with uncertainties. Comparing the quality of registered reports with the quality of regular papers, Soderberg et al. find that registered reports ‘are associated with reviewers perceiving greater rigour and quality without costs on research importance or creativity compared with the standard model’. Papers which follow registered reports still need to undergo peer review since they might exhibit problems (e.g., statistical analysis or unwarranted inferences) which cannot be assessed in advance.

The Quality & Reproducibility school also promotes broadening the scope of peer review beyond research papers, for instance by extending it to data and software. The growing importance of peer review of data is for instance recognized by PLOS (n.d.) with datasets increasingly being peer reviewed alongside papers. PLOS suggests ‘five quick checks’ for peer review of data, claiming that data peer review ‘doesn't have to be time consuming or difficult’. Mayernik et al. (2015) stress that ‘data peer review is not a monolithic concept’, emphasizing the distinction between four scenarios for data peer review: ‘(1) data analyzed in traditional scientific articles, (2) data articles published in traditional scientific journals, (3) data submitted to open access data repositories, and (4) datasets published via articles in data journals’. Peer review of software is still in an early stage of development. A recent development is the introduction of CODECHECK, ‘a system by which the computational workflows underlying research articles are checked’ (Nüst & Eglen, 2021).

A foundational issue for the Quality & Reproducibility school is safeguarding research integrity and identifying scientific misconduct. As discussed by Horbach and Halffman (2018), ‘it is notoriously difficult for peer reviewers to detect cases of intentional data manipulation or fabrication’. However, Horbach and Halffman also point out that ‘one can expect several kinds of questionable research practices … to be detected by reviewers, as in cases of spin, inappropriate use of statistical analysis or data cooking’. They also observe that ‘the use of software tools to detect (self‐)plagiarism, image manipulation and poor statistical analyses has recently increased the detectability of outright misconduct’. Based on a survey of journal editors, Horbach and Halffman (2020) report that text similarity scanners are the most widely adopted innovation in peer review in the last two decades. Various tools are also now commonly deployed by publishers aiming to combat systematic fraud operating through ‘paper mills’, something acknowledged to be an increasing problem, characterized as ‘industrialized cheating’ (Else & Van Noorden, 2021). Many publishers have now publicized their initiatives to respond to this problem, often involving retracting batches of suspicious papers (Oransky, 2022). The STM Integrity Hub is an example of a recent attempt to coordinate work and share good practice in this area across the publisher community (STM, n.d.).

## DEMOCRACY & TRANSPARENCY SCHOOL

The Democracy & Transparency school focuses on making the evaluation of scientific research more open and accountable. Peer review is seen as more democratic when participation in the evaluation of a scientific work is open to a broader group of people (Heesen & Bright, 2020; Tennant et al., 2017). Making peer review more transparent is seen as a way to increase the accountability of editors and peer reviewers, and also to enable information produced in a peer review process to be reused by others, who may find this helpful to develop their own perspective on the strengths and weaknesses of a scientific work.

Throughout the 20th century, a key role in scholarly publishing was reserved for journal editors, who fulfilled important functions, including correspondence with authors, editing of manuscripts, and—where peer review was used in the first place—commissioning reviewers to evaluate manuscripts and interpreting their recommendations (Crane, 1967; Ziman, 2000; Zuckerman & Merton, 1971). Editors make decisions primarily on academic grounds but have also historically been constrained by wider publishing issues, such as page or word limits, or strategies to improve a journal's metrics. While concentration of editorial power has always been considered problematic (Fyfe, 2020; see also Crane, 1967), the model appears to have been relatively uncontested for a long time. In the last two decades, however, editors have increasingly been seen as undemocratic ‘gatekeepers’, and the need for pre‐publication selectivity and filtering less pressing in a digital environment. For instance, according to Tennant and Ross‐Hellauer (2020), ‘editors wield supreme, executive power in the scholarly publishing decision‐making process, rather than it being derived from a mandate from the masses. Because of this, scholarly publishing is inherently meritocratic …, rather than being democratic’. At its core then, this is a question of power within the peer review system, and an attempt to distribute power to a wider range of actors.

Soundness‐only peer review, practiced by open‐access mega‐journals such as *PLOS ONE* and *Scientific Reports*, is probably one of the most influential innovations in peer review in the last two decades. Soundness‐only peer review aims to consider only the soundness or rigour of a manuscript as a basis for acceptance for publication, and to ignore other aspects such as novelty, significance, and relevance. This is an example of the idea of making the evaluation of scientific work more democratic, since judgements about novelty, significance and relevance, normally part of the role of editors and reviewers, are left to ‘the community’. As discussed by Spezi et al. (2018), a key motivation for soundness‐only peer review is that ‘the community is best placed to judge which papers are important and significant—more so than editors and reviewers’. Soundness‐only peer review is ‘sometimes framed as a stance against elitism and the gatekeeping attitude that editors are perceived sometimes to exhibit’. Spezi et al. (2017) interpret the shift towards soundness‐only peer review as ‘a move from the “wisdom of the expert” to the “wisdom of the crowd”’, or alternatively, quoting from a social media debate, as a move from ‘oligarchic pre‐filtering to a democratic post‐filter’ (Spezi et al., 2017). It needs to be recognized, however, that arguments for greater democracy associated with this kind of development, and with other developments promoted by the Democracy & Transparency school, assume a particular community of interested people with sufficient expertise and resources to take part in scholarly discourse—placing implicit limits on the extent of the democracy involved.

Other innovations originating from the Democracy & Transparency school have focused on making peer review more transparent. One way to make peer review more transparent is to publish the reports of peer reviewers, as well as the decision letters of editors and the response letters of authors, alongside published manuscripts. Another way is to publish the identities of peer reviewers. These innovations, typically referred to as transparent peer review or open peer review (Ross‐Hellauer, 2017), have been adopted by an increasingly large number of journals (Wolfram et al., 2020). Publication of reviewer identities was pioneered by *BMJ*: ‘The primary argument against closed peer review is that it seems wrong for somebody making an important judgment on the work of others to do so in secret’. (Smith, 1999). BioMed Central (now called BMC) was another early adopter of open peer review, arguing that ‘open review is ethically superior to anonymous review’ and that it ‘would increase the accountability of the reviewer, giving less scope for biased or unjustified judgments’ (Godlee, 2002). EMBO has also done pioneering work in this area, emphasizing the value of publishing review reports: ‘Referees can be the best writers of published analyses of single papers … So why hide all their incisive, constructive comments, which can remain pertinent even after revision and publication?’ (Pulverer, 2010).

More recent initiatives bring together the ideas of democracy and transparency. *F1000Research* offers immediate publication of a scientific work followed by open soundness‐only peer review, arguing that this ‘publish then filter’ approach ‘removes barriers for readers and authors alike, and it refocuses the role of peer review from, at its worst, a behind‐the‐scenes variety of censorship to, at its best, the process of expert criticism and advice that has always been its core and upon which the progress of science depends’ (Hunter, 2012). In a similar vein, *eLife* announced in 2020 it would ‘replace the traditional “review, then publish” model developed in the age of the printing press with a “publish, then review” model optimized for the age of the internet’ (Eisen et al., 2020). This was followed by the announcement in 2022 that *eLife* would move away from binary accept or reject decisions following peer review, and instead ‘publish every paper it reviews as a Reviewed Preprint, a new type of research output that combines the manuscript with eLife's detailed peer reviews and a concise assessment of the significance of the findings and quality of the evidence’ (eLife, 2022).

Many other individuals and organizations have expressed similar views. Twenty years ago, Godlee (2002) foresaw the system ‘abandoning our current attempts to provide systematic prepublication peer review’. She anticipated the current system being ‘replaced by a system of open commentary and ongoing revision, in which responsibility for quality control is shared by many rather than depending on the necessarily subjective judgments of a chosen few’. Ten years later, a collection of papers in *Frontiers in Computational Neuroscience* presented 18 visions for ‘open evaluation’, offering a variety of approaches for organizing ‘an ongoing post‐publication process of transparent peer review and rating of papers’ (Kriegeskorte et al., 2012). Tennant et al. (2017) presented ‘an overview of what the key features of a hybrid, integrated peer review and publishing platform might be and how these could be combined’, arguing that ‘the major benefit of such a system is that peer review becomes an inherently social and community‐led activity, decoupled from any journal‐based system’. They regard this as ‘a more democratic process’ for knowledge generation. Similarly, Stern and O'Shea (2019) argue for a ‘publish first, curate second’ approach in publishing, emphasizing its greater transparency and focus on ‘peer‐mediated improvement’ of work. This model could be delivered, they argue, through an infrastructure of publishing platforms, publishing potentially high volumes of papers, overlaid by ‘curation journals’, which select already published papers based on their quality.

Some of the above ideas have been implemented by platforms such as ScienceOpen, Peer Community in, PREreview, and various others (O'Sullivan et al., 2021). According to ScienceOpen (2020), ‘peer review suffers from a lack of transparency, recognition, and accountability’. ScienceOpen therefore enables researchers to ‘evaluate and improve the research of their peers’, regardless of any prior peer review that the research may already have undergone. Several platforms enable peer review of preprints (Polka et al., 2022), attempting to organize one of the long‐standing purposes of preprints, of receiving community feedback on papers. An example is Peer Community in, which facilitates the evaluation of preprints ‘based on rigorous peer‐review’ (Peer Community in, n.d.). Following such evaluation, preprints may be recommended, making them “complete, reliable and citable articles, without the need for publication in ‘traditional’ journals”. PREreview is another platform for peer review of preprints. It offers an approach to preprint peer review that is focused on making peer review more equitable and inclusive. Its founders' critique of peer review as practiced by many journals is clear: ‘PREreview … is a direct response to the flawed way scientific research is evaluated. Behind closed doors, a handful of unpaid reviewers—selected opaquely and mainly through personal connections—use subjective criteria to decide the fate of a research article’. (PREreview, n.d.). A recent move, designed as a pragmatic stepping‐stone to make peer review processes more transparent is the Publish Your Reviews initiative, which urges reviewers to make their review reports public alongside a preprint, if one is available (ASAPbio, n.d.).

## EQUITY & INCLUSION SCHOOL

The Equity & Inclusion school emphasizes the need for more equitable, diverse and inclusive approaches to peer review. It promotes a balanced representation of different groups of researchers in the peer review system (in particular in gatekeeping roles as editor or reviewer) in order to create a more diverse and inclusive research system as a whole. The Equity & Inclusion school is especially concerned about biases against researchers based on demographic variables such as gender, geography, race and ethnicity. To promote a balanced representation of different groups of researchers, the *Discussion document: Diversity and inclusivity*, published in 2021 by the Committee on Publication Ethics (COPE, 2021), recommends that ‘editors should be encouraged to actively recruit a diversity of qualified editorial board members and qualified peer reviewers’. The Equity & Inclusion school also stresses the consequences that a lack of diversity among gatekeepers may have for the types of scientific knowledge that are produced and disseminated. In this context, COPE (2021) makes a distinction between ‘knowledge by description’ and ‘knowledge by acquaintance’, with the latter coming ‘from experiencing the phenomenon personally’. They suggest that a lack of diversity among gatekeepers may lead to gaps in knowledge by acquaintance and an incomplete understanding of a phenomenon in the literature.

Another example of a development representative of the Equity & Inclusion school is the work started recently in the context of the *Joint commitment for action on inclusion and diversity in publishing*, an initiative led by the Royal Society of Chemistry and supported by over 50 publishing organizations. These publishers ‘acknowledge the barriers within publishing which authors, editorial decision makers and reviewers from under‐represented communities experience’ (Royal Society of Chemistry, n.d.), and they promise to ‘set minimum targets to achieve appropriate and inclusive representation of … authors, reviewers and editorial decision‐makers’. As part of this initiative, some journals ask researchers to provide information about their gender as well as their race or ethnicity (Else & Perkel, 2022).

In terms of concrete actions, the decision by IOP Publishing to move all its journals from single‐anonymous to double‐anonymous (single‐blind to double‐blind) peer review is noteworthy. According to IOP Publishing (2020), this move is part of its ‘dedication to tackle the significant gender, racial and geographical under‐representation in the scholarly publishing process. Double‐anonymous peer review—where the reviewer and author identities are concealed—has the potential to reduce bias with respect to gender, race, country of origin or affiliation which should lead to a more equitable system’. IOP Publishing seems to be the only publisher in the natural sciences that has adopted double‐anonymous peer review for all its journals. As discussed by Pontille and Torny (2014) and Horbach and Halffman (2018), the social sciences and humanities have a longer tradition in the use of double‐anonymous peer review, reflecting concerns about biases against less prestigious authors and institutions, and against female authors. Recent work on ‘status bias’, with reviewers favouring prominent researchers in their field when author identities are known, has been presented as supporting the case for double‐anonymous peer review (Huber et al., 2022).

Scientific evidence of bias in peer review is, however, limited. Most research has focused on gender bias. Some reports conclude that peer review is indeed biased against female authors. For instance, the Royal Society of Chemistry (2019) states that data for their journals ‘indicates the existence of biases at two review stages: initial assessment by the editor and peer review’. However, this is based on an analysis that does not control for confounding variables. A subsequent large‐scale analysis by Squazzoni et al. (2021) which does control for confounding variables reports no evidence of gender bias. According to Squazzoni et al., their ‘findings indicate that manuscripts submitted by women or coauthored by women are generally not penalized during the peer review process’. Squazzoni et al. also observe that ‘manuscripts by all women or cross‐gender teams of authors had even a higher probability of success in many cases’. In contrast, a number of similar but smaller studies, in the fields of ecology (Fox & Paine, 2019), economics (Card et al., 2020), and microbiology (Hagan et al., 2020) as well as for the journal *eLife* (Murray et al., 2019), present results that do suggest bias in peer review against female authors.

Importantly, the above‐mentioned studies are all observational, making it difficult to provide robust evidence of gender bias. This is acknowledged by Squazzoni et al. (2021), who state that ‘the lack of an objective measure of the quality of manuscripts’ is a major limitation of their work. As pointed out by Lee et al. (2013), this limitation affects most studies reported in the literature: ‘Research on bias as a function of author characteristics adopts the untested assumption that authors belonging to different social categories submit manuscripts … of comparable quality’. Studies that have an experimental instead of an observational design do not suffer from this limitation. In their literature review, however, Lee et al. (2013) report that experimental studies provide no evidence of bias. This is in line with another review of the literature, which concludes that ‘sex discrimination in reviewing of manuscripts … does not in fact exist’ (Ceci & Williams, 2011).

Clearly, bias in peer review remains a contentious issue. Further research is needed in this area, focusing not only on gender but also on other variables, such as geography, ethnicity, and status. Moreover, most work done so far appears to reflect a positivist viewpoint, assuming universal scientific norms. Bringing in complementary social constructivist perspectives, for instance reflecting feminist traditions, may contribute to a richer understanding of issues related to equity and inclusion in peer review.

## EFFICIENCY & INCENTIVES SCHOOL

The Efficiency & Incentives school focuses on streamlining peer review processes and incentivizing participation in them. This school is concerned about the pressure on the peer review system, which makes it increasingly difficult to find peer reviewers, causing the publication of new scientific results to be slowed down. Tennant (2018) quotes estimates that over 2.5 million papers are published each year in English, creating ‘an incredible burden on the global research workforce, considering that a typical research paper requires 2–3 referees and a handling editor, most of whom act on a volunteer basis for scholarly journals’. ‘Reviewer fatigue’ is an increasing problem, especially as ‘evidence suggests that the majority of reviews are performed by a minority of researchers within an increasingly over‐burdened system’ (Tennant, 2018). To reduce the pressure on the peer review system, a large variety of initiatives have been developed to make the system more efficient and to incentivize researchers to contribute to the system.

Portable peer review offers a way to increase the efficiency of peer review. When a manuscript is rejected by a journal and is then submitted to a different journal, portable peer review enables the review reports collected by the former journal to be reused by the latter journal. Publishers have developed workflows to facilitate reuse of review reports by journals within their own portfolio, a process that is sometimes referred to as cascading (Wakeling et al., 2017). In addition, some initiatives have been taken to enable portable peer review across publishers. A pioneering example is the Neuroscience Peer Review Consortium, an initiative launched in 2008 in which neuroscience journals work together to support reuse of review reports: ‘If reviews obtained by one journal could be re‐used by another, a considerable amount of work could be avoided and publication delays could be reduced’. (Neuroscience Peer Review Consortium, n.d.). Individual journals have also developed portable peer review policies. For instance, the editors of *BMC Biology* explicitly invite researchers to send them the review reports of manuscripts rejected by other journals, with the aim to increase the efficiency of peer review (Bell & Kvajo, 2018).

To simplify the implementation of portable peer review, providers of submission systems have started to work together in the Manuscript Exchange Common Approach. Again, the aim is to increase the efficiency of peer review, although technical as well as cultural challenges remain: ‘85% of authors resubmit rejected papers to a different journal. In peer review, papers are often rejected but reviews are not typically shared between journals … 15 million hours of researcher time is wasted each year in reviewing and re‐reviewing unpublished papers’ (Manuscript Exchange Common Approach, n.d.).

Journal‐independent peer review can be seen as a next step in the above developments. An interesting initiative in this area is Review Commons, a collaboration of 17 journals in the life sciences that started in 2019. Like the initiatives mentioned above, Review Commons aims to address the inefficiency of peer review: ‘Journal‐based peer review requires a significant time investment by authors, reviewers and editors, especially when rejected manuscripts are submitted to several journals, each of which in turn starts the review process from scratch’ (Guglielmi, 2021). To increase the efficiency of peer review, the idea of Review Commons is that a manuscript is published on a preprint server and undergoes journal‐independent peer review, after which the authors may decide to submit their revised manuscript, along with the review reports, to one of the journals participating in Review Commons: ‘By allowing reviewers to assess the quality of a study rather than its fit with a specific journal, journal‐independent peer review accelerates and streamlines scientific communication … The ability to transfer referee reports across journals also helps to avoid repeated cycles of peer review, reducing the time that reviewers spend reading and assessing manuscripts’.

The Efficiency & Incentives school has also developed several initiatives to incentivize researchers to perform peer review, typically by giving more visibility and recognition to peer review activities. An important step in this area was the launch of Publons in 2013 (integrated since 2022 into Clarivate's ‘Web of Science’). Like some of the platforms discussed in Section 3, Publons enables researchers to publish reviews (depending on the relevant journal's policy), but in addition it also allows researchers to get recognition for peer review activities that typically remain invisible: ‘Our ultimate aim is to speed up science and improve the slow and inefficient peer‐review process … Publons is not about trying to get people to do more work but about allowing them to get more recognition’ (Johnson, as cited in Research Information, 2014). A similar initiative has been developed by ORCID, enabling researchers to include peer review activities in their ORCID profile. Again, the aim is to incentivize peer reviewers by giving recognition to their work: ‘The time is right to think creatively about how to build incentives (including public acknowledgement) and trust (through validation)’ (Haak & Lawrence, 2015).

There have also been calls to pay peer reviewers for their work as a direct form of recognition. Advocates of this approach argue that it is likely to accelerate the peer review process and improve quality. However, sceptics suggest that with values of $450 per review being suggested as reasonable payment, there is insufficient money in the system to sustain this (Brainard, 2021; Cheah & Piasecki, 2022).

## COMPLEMENTARITIES BETWEEN THE SCHOOLS

When we first discussed the four schools of thought with a few people at senior positions in the publishing industry, one of them immediately claimed that his organization embraces the ideas of all four schools. This raises an interesting question: Is it indeed possible to bring together the ideas of the four schools in a coherent vision on the development of the peer review system? Or are some of the schools based on incompatible ideas, and do individuals and organizations need to choose which of the schools they want to belong to?

To a significant degree, we see the four schools of thought as complementary to each other. One may feel a strong commitment to the ideas of a particular school, but this does not mean that one cannot also be supportive of the ideas of other schools. For instance, someone's primary focus may be on the need to improve the quality of peer review, but at the same time this person may also believe that peer review needs to be made more democratic and more equitable, and this person may also acknowledge that improving the quality of peer review will not be possible without making peer review more efficient. Hence, belonging to a particular school does not necessarily mean that one disagrees with the views of the other schools. It merely means that one chooses to emphasize the importance of the ideas of a specific school.

The complementarity of the different schools of thought is also demonstrated by the way in which innovations in peer review are linked to the schools. While it may be tempting to link each innovation to one specific school, this would yield an oversimplified picture of the landscape of peer review innovation. Many innovations align naturally with the ideas of a particular school, but at the same time they also reflect ideas of other schools.

Open peer review, in which reviewer identities and review reports are published, is a clear example (Ross‐Hellauer, 2017). As discussed in Section 3, open peer review aligns naturally with the ideas of the Democracy & Transparency school. However, by increasing the accountability of peer reviewers, open peer review might also offer a way to improve the quality of review reports (Bruce et al., 2016), which is one of the ambitions of the Quality & Reproducibility school. Likewise, the increased accountability of peer reviewers in open peer review might help reduce biases of peer reviewers against specific groups of authors, in line with the ambitions of the Equity & Inclusion school. Finally, open peer review might incentivize researchers to perform peer review by giving visibility to the work done by peer reviewers. This is one of the ambitions of the Efficiency & Incentives school. Hence, while open peer review may seem most closely aligned with the Democracy & Transparency school, it could potentially help realize some of the ambitions of the other schools as well.

Despite the complementarities of the four schools of thought, some of their ideas do not seem to be compatible. In the next section, we therefore turn to the tensions between the schools.

## TENSIONS BETWEEN THE SCHOOLS

**FIGURE 2:**
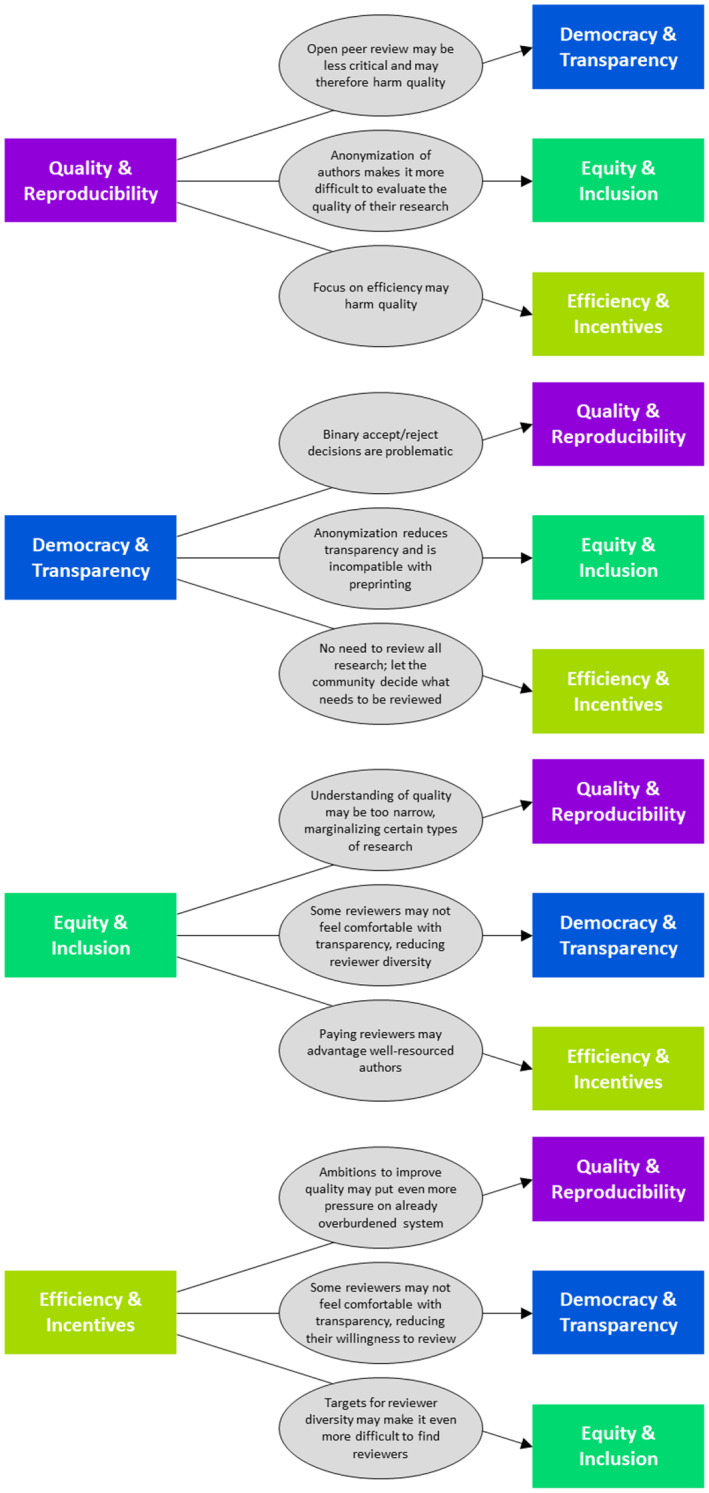
Tensions between the four schools of thought, illustrating where each school may see problems with the others.

Tensions between the various schools of thought pose a challenge because they may lead to conflicting views on how the peer review system can best be improved. Figure 2 shows where the ideas developed by each of the schools are likely to create tensions with the ideas of each of the other schools. For each school, the figure presents a diagram that shows how the school may criticize each of the other schools. The first diagram for instance shows objections of the Quality & Reproducibility school against the other schools. We discuss some of the key tensions between the different schools of thought in this section.

A lot of work in the Quality & Reproducibility school focuses on improving the way in which journals decide which manuscripts to accept for publication and which ones to reject. An example is the work on registered reports, in which a decision on acceptance or rejection is made based on a research plan instead of a complete manuscript that also includes the results of a research project. The Democracy & Transparency school questions the emphasis put on binary accept/reject decisions. Approaches to peer review introduced by this school, based on ideas such as ‘publish, then review’, aim to reduce the importance of these binary decisions or even try to do away with such decisions altogether. This is at odds with many of the ideas of the Quality & Reproducibility school. The latter school, on the other hand, may fear that the open peer review approaches promoted by the Democracy & Transparency school lead to less candid and less critical peer review, which may harm research quality. This may be the case particularly when there is a power asymmetry between a reviewer and an author, for instance when the reviewer is an early career researcher while the author is a prominent senior colleague. The reviewer may then be afraid that a critical non‐anonymous review will have negative career consequences for them.

This tension between the Quality & Reproducibility school and the Democracy & Transparency school relates to the distinction made by Horbach and Halffman (2018) between two perspectives on the scholarly publishing system, referred to as the ‘database frame’ and the ‘library frame’. According to Horbach and Halffman, the database frame ‘seems specifically attractive to those holding realist and positivist views of knowledge’. This frame ‘presents the scientific literature as a database of accurate knowledge or “facts”’. In the library frame, on the other hand, ‘propositions and knowledge claims, as well as their denials, co‐existed in an inter‐textual universe of scientific knowledge claims—some more, some less veracious’. The Quality & Reproducibility school appears to align with the database perspective on the scholarly publishing system, while the Democracy & Transparency school seems more accommodating of the library perspective. These different alignments imply different views of what constitutes valuable and valid contributions to the literature.

The emphasis of the Quality & Reproducibility school on improving the quality of scientific research might arguably result in a somewhat narrow perspective on the notion of quality, causing certain types of research to be favoured over others. This may create tensions with the Equity & Inclusion school. If different social groups prefer to do different types of research, the adoption of a specific notion of quality may be advantageous to some groups and detrimental to others. For instance, if women do more qualitative research than men (Grant et al., 1987), a notion of quality that favours quantitative over qualitative research may cause the work of female researchers to be underrepresented in the literature. This would be worrisome from the perspective of the Equity & Inclusion school.

Conversely, the Quality & Reproducibility school might have concerns about double‐anonymous peer review, which the Equity & Inclusion school seems to prefer over single‐anonymous peer review. To safeguard research quality, the Quality & Reproducibility school may argue that it is important for peer reviewers to know the identity of the authors of a manuscript. This knowledge enables peer reviewers to evaluate a manuscript in the context of earlier work done by the authors and to take into account any conflicts of interest the authors may have.

Double‐anonymous peer review also seems antithetical to the focus on transparency and accountability of the Democracy & Transparency school. While the Democracy & Transparency school may agree with the Equity & Inclusion school that peer review needs to be made more equitable and inclusive, it favours a different approach to reach this goal. Instead of making peer review more anonymous, it prefers to increase the transparency and openness of peer review. As observed by Horbach and Halffman (2018), ‘the issue of reviewer bias as a threat to the quality and fairness of peer review has not only led to the establishment of double‐blind peer review, but also to its radical opposite: the system of open review’. Open peer review increases the accountability of peer reviewers, which may reduce biases (see also Section 6) or at least help to expose them. It may also enable a broader group of researchers to participate in peer review. Furthermore, it is important to recognize that a system of double‐anonymous peer review is incompatible with any forms of dissemination which precede peer review and reveal author identities, including preprinting. It is also very difficult to achieve in practice since researchers will often recognize each other's contributions, especially in small communities.

The challenge of finding a suitable balance between anonymity and transparency is clearly visible in the development of peer review at BMC. As discussed in Section 3, 20 years ago, BMC started to publish the names of peer reviewers, arguing that ‘open review is ethically superior to anonymous review’ and gives ‘less scope for biased or unjustified judgments’ (Godlee, 2002). However, in 2020, BMC reconsidered its open peer review policy. While it still publishes review reports, it no longer mandates publication of reviewer identities and instead prioritizes ‘enhanced author choice and increased diversity within the reviewer pool’ (Hodges, 2020). Another interesting case is IOP Publishing, which recently moved from single‐anonymous to double‐anonymous peer review, as mentioned in Section 4. IOP Publishing argues that anonymity and transparency do not need to be opposites: ‘We will be offering double‐blind review before publication, and transparent review … post‐publication. We believe these two processes complement each other perfectly, allowing for maximum objectivity during the review process, and maximum transparency after publication’. (Eggleton, as cited in Anderson, 2020).

The ambition of the Quality & Reproducibility school to improve research quality may put additional pressure on the peer review system. For instance, increasing the number of peer reviewers per manuscript or extending the scope of peer review to data and software requires additional efforts from peer reviewers. This seems undesirable from the perspective of the Efficiency & Incentives school, which considers the peer review system to be already overburdened. Conversely, the Quality & Reproducibility school may be hesitant about portable peer review, one of the approaches introduced by the Efficiency & Incentives school to increase the efficiency of peer review. To guarantee that published research meets the desired quality standards, journals may prefer to work with their own trusted peer reviewers instead of relying on the judgements of peer reviewers with whom they are not familiar.

Publication of reviewer identities, one of the ideas suggested by the Democracy & Transparency school, may be received with mixed feelings by the Efficiency & Incentives school. As mentioned in Section 6, giving visibility to peer reviewers is a way to recognize their efforts, which may incentivize researchers to perform peer review. However, the opposite effect is also possible. If reviewer identities are published, some researchers, especially those who are in an early career stage or in a vulnerable position, may not feel comfortable writing critical reviews and may therefore be disincentivized to perform peer review. In the case of *BMJ*, it was found that revealing reviewer identities overall has a negative effect on the willingness of researchers to review a manuscript (Van Rooyen et al., 2010).

One of the suggestions made by the Equity & Inclusion school is to increase the diversity of reviewer pools and editorial boards. This may raise some concerns from the viewpoint of the Efficiency & Incentives school. Imposing targets on the diversity of peer reviewers may make it even more challenging to find reviewers. It may also disrupt the gift economy (Kaltenbrunner, Birch, & Amuchastegui, 2022) in which researchers that contribute a lot to a journal, for instance by reviewing large numbers of manuscripts, get recognition by being invited to serve on the journal's editorial board. The Equity & Inclusion school could of course make the counterargument that, despite these short‐term challenges, increasing diversity will in the long term help a journal move towards a more sustainable peer review system.

## CONCLUSIONS

To provide a structured and systematic perspective on the landscape of innovation in peer review, we have suggested that the landscape is shaped by four schools of thought, referred to as the Quality & Reproducibility school, the Democracy & Transparency school, the Equity & Inclusion school, and the Efficiency & Incentives school. These schools have different views on the key problems of the peer review system and the innovations needed to address these problems. While the schools may complement each other, the differences in their views may also give rise to tensions.

We believe that each of the four schools of thought offers a valuable perspective on the challenges faced by the peer review system. To improve peer review, the ideas and ambitions of all four schools need serious consideration. We hope that our work will facilitate conversations between the different schools about the future development of the peer review system. Such conversations may help find creative ways to deal with the tensions between the schools. We expect that all schools will benefit from this. Instead of opposing each other, the schools will strengthen each other.

To bring together the ideas of the four schools of thought, it seems essential to provide room for more heterogeneity in the peer review system. For instance, an option might be to move towards a system in which each scientific work goes through a very basic quality assurance process, which is complemented by the possibility to receive community feedback through some form of open post‐publication peer review. Such an approach would align well with the ideas of the Democracy & Transparency school, and it would also be relatively inexpensive, in line with the efficiency considerations emphasized by the Efficiency & Incentives school. At the same time, for specific scientific works that are considered to be of special interest or importance, the envisioned system could offer a much more in‐depth and rigorous form of peer review, focused on providing robust guarantees of the quality and reproducibility of the research, following the ideas of the Quality & Reproducibility school. This process of in‐depth peer review should be set up in an equitable and inclusive way, taking into account the insights from the Equity & Inclusion school. The envisioned system would also need to leave room for disciplinary variation in the organization of peer review, recognizing that some of the ideas of the various schools may be more relevant in some disciplines than in others.

One of the key factors influencing decisions about peer review models is the extent to which they interact with the recognition and reward systems of the academy. Arguably, approaches to innovation in peer review have been shaped by the fact that peer review, as a mechanism underpinning scholarly publishing, has a profound effect on the status and career progression of researchers. The prestige associated with certain journal titles, fueled by journal impact factors, has often affected the willingness of publishers and editors to experiment with their journals, the motives of those setting up alternatives, and the readiness of authors to engage with different approaches. The role of journals in academic evaluation, however, is now part of a much wider debate on reforms in research assessment. Attempts to move away from crude metrics such as impact factors and to introduce more ‘responsible’ assessment practices are now gaining greater traction, and may reasonably be assumed to affect the willingness of key actors to experiment with new peer review approaches. The drive towards responsible assessment practices also relates to moves in academia towards greater equity, diversity, and inclusion (EDI), something evident in debates around peer review, as we have seen. At the same time, the gathering momentum of open science practices is also affecting the environment, with peer review one component of the research system in which more open ways of working can be adopted. All of these wider developments—new evaluation approaches, EDI initiatives and open science practices—are likely to affect the developments we see in the peer review space as part of a complex interaction.

The connectedness of the peer review system with broader developments in the research system highlights that improving peer review requires coordinated action by a multitude of stakeholders, not only scientific publishers, technology providers, scholarly societies, journal editors and meta‐researchers like ourselves, but also funding agencies, research institutions, governmental organizations and others. Whenever possible, such action should be based on a rigorous evidence‐informed understanding of the peer review system. We hope that the different stakeholders will intensify their efforts to study the peer review system (Rennie, 2016; Squazzoni et al., 2020), to experiment with new forms of peer review, and to introduce improved peer review practices and policies.

## AUTHOR CONTRIBUTIONS

**Ludo Waltman:** Conceptualization; Writing—original draft. **Wolfgang Kaltenbrunner:** Conceptualization; Writing—review and editing. **Stephen Pinfield:** Conceptualization; Writing—review and editing. **Helen Buckley Woods:** Conceptualization; Writing—review and editing.

## FUNDING INFORMATION

This work was supported by the Wellcome Trust [221,297/Z/20/Z], as part of its core funding of the Research on Research Institute (RoRI).

## CONFLICT OF INTEREST STATEMENT

Ludo Waltman is Editor‐in‐Chief of *Quantitative Science Studies*, published by MIT Press. This journal was running a transparent peer review pilot while this paper was being prepared. In the meantime, the pilot has been completed successfully. Ludo Waltman is also coordinator of the ASAPbio Publish Your Reviews initiative, which promotes open peer review practices. Wolfgang Kaltenbrunner, Stephen Pinfield and Ludo Waltman are involved in setting up a new publication platform for research on research.

## Data Availability

No data was used in this research.
